# Interlinkage between inflammation, oxidative stress, and endoplasmic reticulum stress in bisphenols-induced testicular steroidogenesis disturbance: A mini review

**DOI:** 10.18502/ijrm.v23i1.18187

**Published:** 2025-03-21

**Authors:** Nur Erysha Sabrina Jefferi, Asma Afifah Shamhari, Zariyantey Abd Hamid, Siti Balkis Budin, Izatus Shima Taib

**Affiliations:** Centre for Diagnostics, Therapeutics and Investigative Studies, Faculty of Health Sciences, Universiti Kebangsaan Malaysia, Jalan Raja Muda Abdul Aziz, Kuala Lumpur, Malaysia.

**Keywords:** Endocrine disruptors, Endoplasmic reticulum, Inflammation, Oxidative stress, Testicular.

## Abstract

Bisphenols (BP) are endocrine-disrupting chemicals that cause adverse health effects, including testicular steroidogenesis disturbance. Cyclo-oxygenase-2 and nuclear factor erythroid 2-related factor 2 are the target molecules involved in testicular steroidogenesis disturbance via inflammation and oxidative stress (OS), respectively. Interestingly, endoplasmic reticulum (ER) stress was found to be involved in various pathological conditions. However, the mechanisms involved in BP-induced testicular steroidogenesis disturbance remain unclear. Therefore, this research investigates the key mechanisms underlying BP-induced testicular steroidogenesis disturbances. We focus on 3 critical pathways: inflammation, OS, and ER stress. Our findings demonstrate that BP exposure triggers inflammatory responses by targeting the cyclo-oxygenase-2 molecules that impair Leydig cell function. Concurrently, we observed that BP-increased OS via inhibition of nuclear factor erythroid 2-related factor 2, further disrupting steroidogenic enzyme activity. Additionally, ER stress is activated in response to BP exposure, leading to impaired protein synthesis and exacerbating steroidogenic dysfunction. This review elucidates the interlinkage between inflammation, OS, and ER stress in BP-induced testicular steroidogenesis disturbance in which reactive oxygen species is proposed to be the main culprit in linking these 3 mechanisms. These insights provide a crucial foundation for understanding the reproductive toxicology of BPs and inform future strategies for mitigating their effects on male reproductive health.

## 1. Introduction

Numerous toxicants, including endocrine-disrupting chemicals, can impair testicular steroidogenesis and negatively impact human health, particularly the reproductive system (1). Bisphenols (BP) have been utilized in epoxy resin and polycarbonate plastic because of their strong resistance to high temperatures, shatter-resistant properties, and electrical insulation capacity. However, their heavy usage has led to environmental pollution in various areas, including aquatic environments, lands, and air. BPs can be exposed through dermal, ingestion and inhalation (2). Previous studies have shown that BPs affect human health by targeting the endocrine system, disrupting the hypothalamus-pituitary-gonad axis and testicular steroidogenesis, which negatively impacts the development and functionality of Leydig cells (3, 4).

Inflammation and oxidative stress (OS) are well-known mechanisms involved in pathological conditions induced by exogenous toxicants (5, 6). Inflammation can be defined as a mechanism by which the immune system identifies and eliminates detrimental agents. Meanwhile, OS is defined as the imbalance between reactive oxygen species (ROS) and antioxidant defenses. BPs trigger inflammation and OS by targeting the cyclo-oxygenase-2 (COX-2) (7) and nuclear factor erythroid 2-related factor 2 (Nrf2) molecules (8), respectively. Current studies have revealed that endoplasmic reticulum (ER) stress is also involved in the testicular steroidogenesis disturbance induced by toxicants (9). ER stress refers to a state in which the ER, a cellular organelle in charge of protein folding, lipid production, and calcium storage, becomes overworked or dysregulated (10). Initially, the ER stress will result in cell protection by restoring and maintaining ER homeostasis. If ER stress persists or becomes too severe, it can trigger apoptotic cell death by activating pro-apoptotic pathways (9).

Testicular steroidogenesis is essential for male fertility, influencing sperm production, libido, and overall reproductive function. Disruptions in this process can lead to various health issues, including infertility, hormonal imbalances, and developmental disorders (11). The mechanisms by which BPs induce these disturbances are complex and multifaceted. Therefore, understanding the key mechanisms behind BP-induced disturbances in testicular steroidogenesis is crucial for developing effective strategies to mitigate their impact on male reproductive health. This article explores the linkage between inflammation, OS, and ER stress in BPs-induced testicular steroidogenesis disturbance, shedding light on potential preventive and therapeutic approaches.

## 2. BPs-structure and modifications

BPs are environmental phenolic compounds consisting of 2 linked phenols. They are primarily found in polycarbonate plastics used in consumer goods (12). BPA, first produced in 1891 and used in plastic manufacturing since the 1950s, is particularly concerning due to its potential to contaminate the environment, as it is present in air, water, soil, sediment, indoor dust, and human tissues (13, 14). BPA has been classified as an environmental contaminant and an endocrine-disrupting chemical harmful to both human health and wildlife (15, 16).

Even in low doses, BPA can disrupt hormonal synthesis and metabolism by mimicking or inhibiting hormone receptors. In humans, elevated BPA levels are linked to the onset and progression of various health issues (17, 18). Several countries have prohibited the use of BPA in a range of consumer goods (19). Therefore, the scientific community has begun to focus more on their research interest in finding the best analogs to replace BPA. The commonly used BPA analogs are BPB, BPE, BPS, BPP, BPZ, BPAP, BPAF, BPF, and tetramethyl BP which have been utilized in manufacturing next-generation of BPA-free products (20).

Various BPA analogs have been developed to achieve specific material properties by adding extra functional groups with specific bridge bonds. BPB is commonly found in polymers used for food containers (21). BPE, with its unique structure, enhances mechanical properties (22). BPS is suitable for products that require higher heat resistance and reduced color (23). BPF is appropriate for certain epoxy resins and coatings (24). BPAF exhibits exceptional thermal and chemical stability, making it ideal for high-performance applications such as aircraft coatings and specialized packaging for high-temperature food (25). BPAP is vital in polycarbonate plastics and epoxy resins, used in food containers (20). BPZ offers high thermal stability and transparency, making it suitable for clear plastic packaging (21, 24). BPP increased heat stability for specialty food packaging (24). Last, tetramethyl BP is utilized as a flame retardant (25). The commonly commercialized BP analogs with their physical properties are shown in table I.

**Table 1:** Physical properties of commonly commercialized bisphenol analogs

**Compound**	**Abbreviations**	**IUPAC name**	**Molecular formula**	**Molecular weight (g/mol)**	**Melting point ( C)**
**Bisphenol A**	BPA	4,4-(propane-2,2-diyl) diphenol	C_15_H_16_O_2_	228.29	158–159
**Bisphenol AF**	BPAF	4-[2-(4-hydroxyphenyl) propan-2-yl] phenol	C_22_H_16_F_4_O_2_	386.35	186–190
**Bisphenol AP**	BPAP	4,4-(1-Phenyl-1,1-ethanediyl) diphenol	C_24_H_20_O_4_	372.41	250–252
**Bisphenol B**	BPB	4-[2-(4-hydroxyphenyl) butan-2-yl] phenol	C_15_H_14_O_2_	226.27	157–159
**Bisphenol C**	BPC	4-[2,2-dichloro-1-(4-hydroxyphenyl) ethenyl] phenol	C_15_H_12_O_2_	224.25	145–146
**Bisphenol E**	BPE	4,4-(1,1 ethanediyl) diphenol	C_14_H_14_O_2_	214.26	123–127
**Bisphenol F**	BPF	4-[(4-hydroxyphenyl) methyl] phenol	C_13_H_12_O_2_	200.23	130–132
**Bisphenol G**	BPG	4,4'-(1-methylethylidene) bis (2-(1 methylethyl)	C_19_H_20_O_4_	312.36	164–165
**Bisphenol P**	BPP	4,4-(1,4-phenylenedi-2,2-propanediyl) diphenol	C_21_H_18_O_4_	326.36	182–183
**Bisphenol PH**	BPPH	5,50-(2,2-propanediyl)di(2-biphenylol)	C_24_H_22_O_4_	374.43	220–222
**Bisphenol S**	BPS	4,40-(propane-2,2-diyl)diphenol	C_12_H_10_O_4_S	250.27	155–160
**Bisphenol Z**	BPZ	4-[1-(4-hydroxyphenyl)cyclohexyl] phenol	C_23_H_18_O_2_	326.39	180–182
IUPAC: International Union of Pure and Applied Chemistry					

## 3. Testicular steroidogenesis 

Steroidogenesis is primarily regulated by the testis, where Leydig cells produce testosterone, which is crucial for fetal growth and male development. This hormone is necessary for spermatogenesis and maintaining secondary sexual functions, with intratesticular testosterone levels being nearly 100 times higher than in the bloodstream (26). The process of testosterone biosynthesis involves several signaling pathways and steroid hormones (15, 27). Testicular steroidogenesis is a complex process that converts cholesterol into active steroid hormones. It is influenced by 2 main factors: the availability of cholesterol and the levels of steroidogenic enzymes (16). The hypothalamic-pituitary-gonadal axis regulates this process. The hypothalamus releases gonadotropin-releasing hormone, prompting the pituitary gland to release luteinizing hormone. Luteinizing hormone binds to receptors on Leydig cells, activating pathways that increase cyclic adenosine monophosphate levels, which then activate protein kinase A. Protein kinase A phosphorylates the steroidogenic acute regulatory (StAR) protein, enabling cholesterol transport (28).

Cholesterol, essential for steroidogenesis, can be synthesized in cells or obtained from circulating lipoproteins like high-density lipoprotein. The scavenger receptor class B type 1 plays a key role in cholesterol uptake and balance (27). Cholesterol is transported to mitochondria through vesicular and nonvesicular pathways, facilitated by the transduceosome complex, which then converts cholesterol to pregnenolone via cytochrome P450 (CYP) 11A1 (29). Pregnenolone then moves from the mitochondria to the ER via specialized membrane regions called mitochondria-associated membranes (30). In the ER, hydroxysteroid dehydrogenases (HSD) and CYP enzymes convert pregnenolone to testosterone primarily through the Δ 5 pathway. This process involves several steps: pregnenolone is converted to 17α-hydroxypregnenolone by CYP17A1, then to dehydroepiandrosterone, and finally to androstenediol, with testosterone being produced by the action of 3β-HSD. Testosterone can also be converted to dihydrotestosterone by 5α-reductase or to estrogens via CYP19A1 (15, 16).

## 4. Results and Discussion

**Figure 1:**
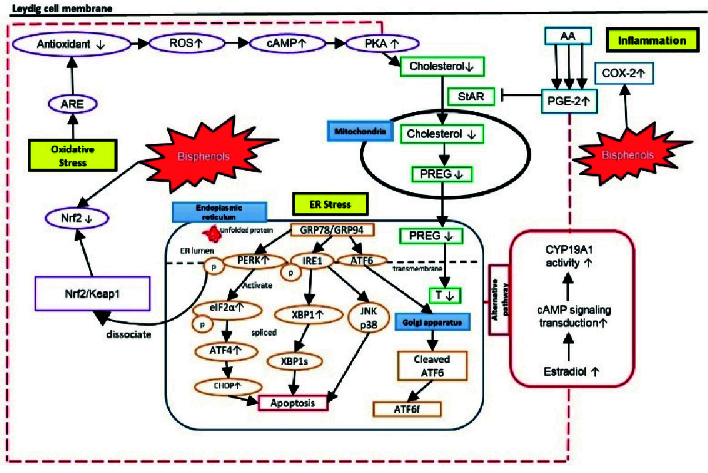
Inflammation, OS, and ER stress in BP-induced steroidogenesis disturbances. Exposure toward BP causes inflammation and OS by targeting COX-2 and Nrf2, respectively. Meanwhile, BPs induce ER stress by triggering unfolded protein response signaling via IRE1, PERK, and ATF6. In the ER, GRP78, and GRP94 are sequestered by misfolded proteins, which trigger IRE1 to cleave XBP1 mRNA and cause apoptosis. PERK phosphorylates eIF2α, selectively inducing ATF4, leading to apoptosis. ATF6 is translocated into the Golgi apparatus, cleaved by protease, and released, leading to apoptosis. PERK also activates Nrf2 by dissociating Nrf2 from Keap1. BPs cause OS by reducing the production of Nrf2, leading to decreased antioxidant responses due to increased ROS production in the Leydig cells. This leads to increased cAMP and PKA, which limits cholesterol transportation into mitochondria. Meanwhile, BP caused inflammation via increased expression of COX-2 and this caused more AA to be converted into PGE-2. The increased level of PGE-2 and the reduced level of PKA inhibited StAR from transporting cholesterol into mitochondria, leading to decreased testosterone synthesis. At the same time, an alternative pathway is activated via increased CYP19A1 activity by increasing cAMP signaling transduction and converting more testosterone into estradiol, leading to dysregulation of testicular steroidogenesis. AA: Arachidonic acid, ARE: Antioxidant response element, ATF4: Activating transcription factor 4, ATF6: Activating transcription factor 6, ATF6f: Activating transcription factor 6 fragment, cAMP: Cyclic adenosine monophosphate, CHOP: C/EBP homologous protein, COX-2: Cyclo-oxygenase-2, CYP19A1: Cytochrome P450 19A1 (aromatase), ER: Endoplasmic reticulum, eIF2α: Eukaryotic initiation factor-2α, GRP78: Glucose-regulated protein 78, GRP94, glucose-regulated protein 94, IRE1: Inositol-requiring enzyme 1, JNK: c-Jun N-terminal kinase, Keap1: Kelch-like ECH-associated protein 1, Nrf2: Nuclear respiratory factor 2, OS: Oxidative stress, PGE-2: Prostaglandin 2, PKA: Protein kinase A, PREG: Pregnenolone, PERK: Protein kinase RNA-like ER kinase, p38: p38 mitogen-activated protein kinase, ROS: Reactive oxygen species, StAR: Steroidogenic acute regulatory protein, T: Testosterone, XBP1: X-box binding protein 1.

BPs trigger inflammatory and stress responses in testicular steroidogenesis, primarily disrupting this process through inflammatory and OS pathways (15). However, data on their effects on ER stress remain limited. BPs act as inflammatory stimuli, activating cascades that disrupt steroidogenesis. Notably, COX-2 is a key target in BP-intoxicated rats, where its increased expression leads to elevated prostaglandin E2 (PGE-2) formation (31–34), ultimately enhancing estradiol synthesis (35). Exposure to BPs also induces stress signals in Leydig cells, resulting in ROS formation in mitochondria. This overwhelms endogenous antioxidant defenses, as indicated by reduced levels of superoxide dismutase (SOD), reduced glutathione (GSH), and catalase (CAT), along with increased malondialdehyde levels in BP-induced testicular disturbances (36–40). ER stress is also implicated in testicular steroidogenesis disturbances. In response to ER stress, cells initiate the unfolded protein response (UPR) to restore homeostasis or trigger apoptosis in severe cases. Yet only 2 studies in the past 5 yr have reported on ER stress related to BP-induced steroidogenesis disturbances, highlighting a significant research gap (41, 42). The details of hypothesized mechanisms involved in testicular steroidogenesis disturbance induced by BPs are discussed in figure 1.

### BPs targeted COX-2 in inflammatory pathways

The inflammatory response can be summarized in 4 key steps: 1) recognition of harmful stimuli by cell surface pattern receptors; 2) activation of inflammatory pathways; 3) recruitment of inflammatory cells; and 4) production of inflammatory markers (43). COX-2 is an important inflammatory marker involved in cell responses, proliferation, apoptosis, and various pathological processes, including ferroptosis, an iron-dependent form of cell death. COX-2 has been found as one of the targeted molecules in BPs-intoxicated rats (31–34). The increased expression of COX-2 eventually caused an increase in the PGE-2 formation, one of the molecules involved in the activation of alternative pathways in testicular steroidogenesis increasing estradiol synthesis (35).

Recent findings found that dysregulation of COX-2 expression could be induced by BP exposure. To the best of our knowledge, over the past 5 yr, studies investigating the association between COX-2 and BP-induced testicular steroidogenesis disturbance remain limited. A recent study found that COX-2 mRNA expression levels increased in mouse testicular tissues exposed to 50 mg/kg/bw and 100 mg/kg/bw of BPA, indicating that BPA exposure can alter the expression of ferroptosis-related genes in the testis through inflammatory mechanisms (31). The COX-2 mRNA expression was found to be upregulated together with reduced testosterone levels in rat testicular Leydig R2C cells treated with BPA (32). Moreover, nuclear factor kappa-light-chain-enhancer of activated B cells-COX2 and p38 Mitogen-activated protein kinases-COX2 signaling pathways were found to be associated with oxidative damage and chronic inflammation in aging, which limit testosterone synthesis. This further supported recent findings which demonstrated increased production of COX-2 and nuclear factor kappa-light-chain-enhancer of activated B cells followed by reduced testicular testosterone level (44, 45) as well as decreased serum testosterone level (46). In contrast, a significant increase in testosterone levels was found in BPA and BPS exposure even though the expression of COX-2 in testicular tissues was significantly high (33, 47).

These uncertain results of testosterone levels in BPs-induced testicular steroidogenesis indicated that BPs could exhibit endocrine-disrupting effects by interfering with testosterone homeostasis (25). It is postulated that the BP-induced inflammation increases the COX-2 expression, which aids arachidonic acid in the formation of PGE-2 in testicular steroidogenesis. Besides, the increased formation of PGE-2 is involved in the negative feedback in testicular steroidogenesis by inhibiting cholesterol transportation by StAR into the ER. A decrease in mRNA expression of COX-2 in prenatal BPA-exposed offspring testis could affect PGE-2 synthesis in the testis (35), leading to testicular steroidogenesis disturbances (15, 28). Table II summarizes the findings on BP-induced inflammation and its effect on testicular steroidogenesis from in vivo models.

**Table 2:** Bisphenol-induced inflammation evidence in testicular steroidogenesis from in vivo model

**Author, year (Ref)**	**Type of bisphenol**	**Dosage**	**Duration of exposure**	**Animal model/cell line**	**Findings** **(inflammation)**	**Findings (steroidogenesis)**
**Li ** ***et al.*** **, (31)**	BPA	0.5, 10, 50, 100 and 200 mg/kg bw	45 days	8-wk-old specific pathogen-free male mice	COX-2 mRNA expression	NA
**Molangiri ** ***et al.*** **, (33)**	BPA and BPS	0.4, 4, 40 ug/kg bw of BPA and BPS	GD4-GD21	Male offsprings of pregnant Wistar rats	↑ COX-2	↓ HSD11β2, ↓ HSD17β7
**Kumar ** ***et al.*** **, 2021 (47)**	BPS	75 mg/kg body bw	28 days	Adult golden hamster, aged 90–100 days (Testis)	↑ NF-κB, ↑ COX-2	↓ Serum T
**Tekin ** ***et al.*** **, 2024 (45)**	BPA	50 mg/kg and 100 mg/kg	14 days	Adult male Sprague Dawley rats, aged 12 wk (Testis)	↑ TNF-α, ↑ IL-1β, ↑ IL-6, ↑ COX-2, ↑ NF-κB, ↓ IL-10	↓ Testicular testosterone, ↓ 17β-HSD3, ↓ StAR ↓ P450scc
**Tekin ** ***et al.*** **, 2022 (44)**	BPA	100 mg/kg	14 days	10-wk-old Sprague Dawley male rats (Testis)	↑ TNF-α, ↑ IL-1β, ↑ IL-6, ↑ COX-2, ↑ NF-κB, ↓ IL-10	↓ Testicular testosterone, ↓ 17β-HSD3, ↓ StAR ↓ P450scc
**Sahu ** ***et al.*** **, 2023 (46)**	BPS	150 mg/kg	28 days	Adult male Parkes strain mice, aged 90 days	↑ TNF-α, ↑ IL-1β, ↑ IL-6, ↑ COX-2, ↑ NF-κB, ↓ IL-10	↓ Serum T
↑ : Increased, ↓ : Decreased, µg/kg: Micrograms per kilogram, BPA: Bisphenol A, BPS: Bisphenol S, bw: Body weight, COX-2: Cyclooxygenase-2, GD: Gestational day, HSD11β2: 11-beta-hydroxysteroid dehydrogenase type 2, HSD17β7: 17-beta-hydroxysteroid dehydrogenase type 7, IL-1β: Interleukin-1 beta, IL-6: Interleukin-6, IL-10: Interleukin-10, mRNA: Messenger RNA, mg/kg: Milligrams per kilogram, NA: Not applicable, NF-κB: Nuclear factor kappa B, StAR: Steroidogenic acute regulatory protein, T: Testosterone, TNF-α: Tumor necrosis factor-alpha, 17β-HSD3: 17-beta-hydroxysteroid dehydrogenase type 3, P450scc: Cytochrome P450 side-chain cleavage						

### BPs targeted Nrf2 in OS pathways 

The inflammation process has been reported to mediate OS in Leydig cells (48). During OS, redox homeostasis has been disturbed. Redox equilibrium is maintained by internal and extracellular buffering mechanisms, involving small molecule and protein-based buffers including GSH/GSH disulfide, cysteine/cystine, and oxidized/reduced thioredoxin (49). Besides, antioxidant enzymes such as SOD, CAT, GSH peroxidase, and thioredoxin reductase, as well as nonenzymatic antioxidants like α-tocopherol (vitamin E), ascorbate (vitamin C), β-carotene, and flavonoids, also helps in maintaining the redox equilibrium in cell (50). An excessive increase of ROS, together with an inadequate antioxidant defense or failure of the cellular buffering system will lead to OS (51). OS induced by BPs leads to a reduced capacity of antioxidant defense to detoxify ROS in cells and tissues.

Previous studies reported that BPs induced OS in Leydig cells after in vivo and in vitro exposures (36–39, 42, 44–46, 52–57). OS occurs in the testis of sexually matured mice, proven by increased malondialdehyde levels and decreased antioxidant levels after exposure to BPA at the dose of 50 mg/kg (42). Repeated exposure to BPA for 35 days decreased testosterone synthesis via OS mechanisms (57). Interestingly, BPA exposure in adult male Sprague-Dawley rats for 14 days also caused OS in the testis, which ultimately led to a reduction in testosterone synthesis, as evidenced by the inhibition of several enzymes involved in testicular steroidogenesis (44, 45). Furthermore, by increasing the mRNA levels of genes linked to OS, it was discovered that OS contributed to BPs-induced cell death in TM3 Leydig cells (36, 39). Previous studies also reported that the effects of BPS after 28 days of exposure also caused OS leading to decreased in testosterone level (46, 47). Moreover, in vitro exposure to BPS at the doses of 100, 200, and 400 μM for 48 hr resulted in cytotoxicity of Leydig cell lines due to OS evidenced by elevated ROS levels, SOD, and CAT levels, and mitochondrial dysfunction (36). Furthermore, exposure to BPF at concentrations of 20, 40, and 80 μM for 72 hr caused an increase in ROS production in TM3 Leydig cells in a dose-dependent manner (43).

Exposure to Tetramethyl Bisphenol A (TMBPA) at the highest dosage, which is 200 mg/kg for 21 days, triggered the OS to occur in the Leydig cell of preadolescent Sprague-Dawley rats. This BPA analog not only lowers the GSH level and GSH/GSH disulfide ratio, but it also downregulates genes like *Sod1, Sod2,* and *Cat* that are involved in the generation of antioxidants (37). BPAF at the same dose as TMBPA also decreased the SOD2 activity and increased the formation of ROS in ethane dimethane sulfonate deactivated-Leydig cells, showing that OS occurs after 7–28 days of post-ethane dimethane sulfonate exposure (40). Only one study reported on the in vitro exposure of TMBPA-induced OS in Leydig cells, isolated from 35–60-day-old Sprague Dawley rats (38).

OS may also activate other nuclear transcription factors known as Nrf2. It is normally attached to Kelch-like ECH-associated protein 1 (Keap1), its inhibitor protein, which directs Nrf2's proteasomal destruction. Nevertheless, Nrf2 dissociates from Keap1 in reaction to electrophilic substances or OS, and it moves to the nucleus, where it attaches to the antioxidant response element in the promoter regions of its target genes (58). Transcription of these target genes is involved in controlling the OS and inflammation by scavenging the ROS, eliminating electrophilic toxicants, maintenance of GSH homeostasis and redox balance, suppressing pro-inflammatory signals, and potentiation of anti-inflammatory signaling (59).

Exposure to BPF decreased the Nrf2 in the TM3 Leydig cell line. TMBPA downregulates the Keap1 and Nrf2, resulting in OS (39). Nrf2 was found to be increased in the cytoplasm and decreased in the nucleus of a Swine testis cell exposed to BPA at the concentration of 125 nM for 24 hr, triggering OS to occur due to the inability to produce the antioxidants (53). BPA was also found to induce testicular OS by decreasing the Nrf2 after neonatal exposure during postnatal day 1–35 (60). Meanwhile, exposure to BPS at the dose of 2 and 200 mg/kg induced OS via inhibition of Nrf2 and upregulated the Kaep1 in the testis of adult male mice (42). Table III summarizes the findings on BP-induced OS and its effect on testicular steroidogenesis from in vivo models and in vitro models.

**Table 3:** Bisphenol-induced OS evidence in testicular steroidogenesis from in vivo and in vitro models

**Author, year (Ref)**	**Type of bisphenol**	**Dosage**	**Duration of exposure**	**Animal model/cell line**	**Findings** **(OS)**	**Findings (steroidogenesis)**
**Molangiri ** ***et al.*** **, 2022 (33)**	BPA and BPS	0.4, 4, 40 ug/kg bw of BPA and BPS	GD4-GD21	Male offsprings of pregnant Wistar rats	↑ MDA	↓ HSD11B2, ↓ HSD17B7
**Qi ** ***et al.*** **, 2024 (42)**	BPA	50 mg/kg	30 days	Sexually mature Kunming male mice (n = 50; body weight: 35 gr ± 2 gr; age: 2 months old)	↑ MDA, ↓ SOD1, ↑ SOD2, ↑ CAT, ↑ GSH	↓ StAR, ↓ CYP11A1, ↓ CYP17A1, ↓ 17β-HSD, ↑ CYP19A1
**Chen ** ***et al.*** **, 2022 (53)**	BPA	125 nM	24 hr	Swine testis cell	↑ Nrf2 (cytoplasm), ↓ Nrf2 (nucleus)	NA
**Ling ** ***et al.*** **, 2023 (60)**	BPA	0, 0.1, and 5 mg/kg	PND 1–35	Neonatal male mice	↓ Total antioxidant capacity, Nrf2, NQO1, and GPx-1 of testes	NA
**Tekin ** ***et al.*** **, 2024 (45)**	BPA	50 mg/kg and 100 mg/kg	14 days	Adult male Sprague Dawley rats, aged 12 wk (testis)	↑ MDA, ↓ SOD, ↓ CAT, ↓ GSH, ↓ GPx, ↓ Nrf2, ↓ HO-1	↓ Testicular testosterone, ↓ 17β-HSD3, ↓ StAR, ↓ P450scc
**Tekin ** ***et al.*** **, 2022 (44)**	BPA	100 mg/kg	14 days	10-wk-old Sprague Dawley male rats (testis)	↑ MDA, ↓ SOD, ↓ CAT, ↓ GSH, ↓ GPx	↓ Testicular testosterone, ↓ 17β-HSD3, ↓ StAR, ↓ P450scc
**Khazaeel ** *** et al.*** **, 2022 (57)**	BPA	5 mg/kg, 600 mg/kg	35 days	Adult male mice, weighing 25–30 gr	↓ GPx, ↓ CAT, ↓ SOD, ↑ MDA	↓ Serum T
**Zhang ** ***et al.*** **, 2022 (36)**	BPS	100, 200, and 400 μM	48 hr	TM3 mouse Leydig cells	↓ SOD, ↓ CAT, ↑ ROS	NA
**Sahu ** ***et al.*** **, 2023 (46)**	BPS	150 mg/kg	28 days	Adult male Parkes strain mice, aged 90 days	↑ MDA, ↓ SOD, ↓ CAT, ↓ MT-1, ↓ SIRT-1, ↓ PGC-1α, ↓ FOXO-1, ↓ Nrf2, ↓ HO-1	↓ Serum T
**Kumar ** ***et al.*** **, 2021 (47)**	BPS	75 mg/kg body weight/day	28 days	Adult golden hamster, aged 90–100 days (Testis)	↓ SOD, ↓ CAT, ↑ MDA, ↓ Nrf2, ↓ HO-1	↓ Serum T
**Wang ** ***et al.*** **, 2023 (56)**	BPS	2, 20, and 200 mg/kg	28 days	Adult male mice C57BL/6 (testis)	↓ CAT, ↓ SOD, ↓ GPx, ↓ Nrf2, ↑ Keap1	↓ Testosterone, ↓ StAR, ↓ CYP11A1, ↓ CYP17A1, ↓ 3β-HSD, ↓ 17β-HSD
**Zhou ** ***et al.*** **, 2023 (39)**	BPF	20, 40, and 80 μM	24, 48, and 72 hr	TM3 cells	↑ ROS, ↓ Nrf2	NA
**Odetayo ** ***et al.*** **, 2023 (4)**	BPF	30 mg/kg	28 days	Wistar rats (160–180 gr) (Testis)	↓ CAT, ↓ SOD, ↓ GSH, ↓ GST, ↓ GPx, ↓ Nrf2, ↑ MDA	↓ 3β-HSD, ↓ 17β-HSD
**Tian ** ***et al.*** **, 2022 (38)**	BPAF	10, 50, and 200 mg/kg/d	GD 14 to 21	Pregnant Sprague-Dawley rats (Isolate Leydig cells of neonatal rats at day 1)	↓ SOD1, ↓ SOD2 ↓ CAT, ↑ MDA	↓ Serum T, ↓ Expression of StAR, Cyp17a1, Hsd17b3
**Hu ** ***et al.*** **, 2022 (37)**	TMBPA	0, 10, 100, and 200 mg/kg body weight 0, 1, 10, and 50 μM TMBPA	21 days 24 hr at 34 C	35-day-old male Sprague-Dawley rats Leydig cells isolated from 35- and 56-day-old SD rats	↓ GSH, ↓ GSH/GSSG ratio,↑ MDA downregulate: Sod1, Sod2, Cat, Keap1, Nrf2 ↑ ROS; ↓ Mitochondrial membrane potential	↓ Serum T
↑ : Increased, ↓ : Decreased, C: Degree celsius, μM: Micromolar, µg/kg: Micrograms per kilogram, BPA: Bisphenol A, BPAF: Bisphenol AF, BPF: Bisphenol F, BPS: Bisphenol S, bw: Body weight, CAT: Catalase, Cat1: Gene encoding catalase, CYP11A1: Cytochrome P450 11A1, CYP17A1: Cytochrome P450 17A1, Cyp17a1: Gene encoding cytochrome P450 17A1, CYP19A1: Cytochrome P450 19A1 (aromatase), FOXO-1: Forkhead box o1, g: Gram, GD: Gestational day, GPx: Glutathione peroxidase, GSH: Glutathione, GSSG: Glutathione disulfide, GST: Glutathione transferases, h: Hour, HO-1: Heme oxygenase-1, Hsd17b3: Gene encoding hydroxysteroid 17-beta dehydrogenase 3, HSD11β2: 11-beta-hydroxysteroid dehydrogenase type 2, HSD17β7: 17-beta-hydroxysteroid dehydrogenase type 7, Keap1: Kelch-like ECH-associated protein 1, MDA: Malondialdehyde, MT-1: Metallothionein, mg/kg: Milligrams per kilogram, mg/kg/d: Micrograms per kilogram per day, NA: Not applicable, NQO1: NAD(P)H quinone dehydrogenase 1, nm: Nanomolar, Nrf2: Nuclear respiratory factor 2, OS: Oxidative stress, PGC-1α: Peroxisome proliferator-activated receptor gamma coactivator 1-alpha, PND: Postnatal day, ROS: Reactive oxygen species, P450 scc: Cytochrome P450 side-chain cleavage, SD: Sprague Dawley, SIRT-1: Silent information regulator 1, SOD: Superoxide dismutase, SOD: Superoxide dismutase, gene encoding StAR: Steroidogenic acute regulatory protein, T: Testosterone, TMBPA: Tetramethylbisphenol A, 3β-HSD: 3β-hydroxysteroid dehydrogenase, 17β-HSD: 17-beta-hydroxysteroid dehydrogenase						

### BPs-induced ER stress

The ER is a vital organelle involved in protein folding, lipid synthesis, calcium balance, and steroid hormone production (61). When ER homeostasis is disrupted, it triggers the UPR, leading to ER stress. Under normal conditions, both glucose-regulated proteins (GRP) 78 and 94 bind to UPR stress detectors (protein kinase RNA-like ER kinase [PERK], inositol-requiring enzyme 1 and activating transcription factor 6), keeping them inactive. These proteins help with proper protein folding and prevent the buildup of misfolded proteins. During ER stress, GRP78 and GRP94 dissociate from these detectors, allowing them to activate through phosphorylation and re-localization, which initially protects the cell by restoring ER balance. However, prolonged ER stress can result in cell apoptosis through the activation of C/EBP homologous protein (CHOP), c-Jun N-terminal kinase, and caspases (9).

ER stress can occur in Leydig cells, which can lead to reproductive toxicity and male infertility. One important aspect of ER stress is its involvement in the regulation of steroidogenesis, the process by which steroid hormones are synthesized (30). Testosterone is among the steroid hormones that are synthesized in the smooth ER of Leydig cells. In normal physiological conditions, GRP78 is needed to fold the StAR protein which is pivotal for cholesterol transportation from the outer mitochondrial membrane to the inner mitochondrial membrane for testosterone synthesis. Therefore, the absence of GRP78 causes the unfolding of StAR, leading to the proteolyzation of this protein. However, during ER stress, the upregulation of GRP78, PERK, and CHOP causes the downregulation of StAR protein and 3β-HSD enzymes. In addition, 3β-HSD was also found to be downregulated upon activation of the activating transcription factor 6 pathway.

It has been demonstrated that BPs interfere with ER function by inducing ER stress in Leydig cells. Tetrachlorobisphenol A had been reported to induce ER stress in Leydig cells in vitro, proven by an increase in GRP78 level. Besides, tetrachlorobisphenol A also causes eukaryotic translation initiation factor 2 subunit alpha phosphorylation, leading to increased formation of activating transcription factor 4, a protein regulated by eukaryotic translation initiation factor 2 subunit alpha (41). BPA also triggered ER stress in the testicular tissue of male mice after 30 days of exposure, proven by overexpression of GRP78, phosphorylated PERK, phosphorylated eukaryotic translation initiation factor 2 subunit alpha, p-inositol-requiring enzyme 1, X-box binding protein 1 and activating transcription factor 6 (42). Furthermore, a previous study also found that BPA-induced ER stress on mouse spermatocytes GC-2 cells and adult mice. The researchers conclude that the ROS-regulated PERK/eukaryotic translation initiation factor 2 subunit alpha/CHOP pathway is crucial in the male reproductive toxicity induced by BPA (62).

Interestingly, BPA and its analogs, BPF and BPS, were found to cause an increase in the expression of genes related to ER stress in PNT1A cells, a normal human prostate epithelium cell. However, only BPA has been found to increase the ROS levels in PNT1A cells. In addition, the presence of BPA also caused an increase in the production of the CHOP/DNA damage-inducible transcript 3 protein, which is associated with ER stress, in PNT1A cells (63). Only 2 studies have reported on the effects of BP-induced testicular steroidogenesis disturbance via ER stress (41, 42). This limitation portrays that ER stress could be the potential underlying mechanism to be investigated in future for the other factors that are involved in BPs-induced testicular steroidogenesis disturbance. Table IV summarized the findings on BP-induced ER stress and its effect on testicular steroidogenesis from in vivo and in vitro models.

## 5. Proposed interlinkage between inflammation, OS, and ER stress mechanisms

The interplay between inflammation, OS, and ER stress creates a feedback loop that amplifies cellular damage including testicular steroidogenesis disturbance. Inflammatory mediators, such as cytokines and chemokines, can trigger both OS and ER stress. The ROS generated during OS can damage the ER, causing protein misfolding and activating the UPR. This activation of the UPR, in turn, induces inflammatory pathways. Meanwhile, it is proposed that if the inflammation process is persistent, this will eventually exacerbate the OS and ER stress, leading to a vicious cycle of tissue damage and cell death, thus reducing the ability of Leydig cells to synthesize testosterone.

It is also hypothesized that the localization of specific organelles could also be the contributing factor for these mechanisms influencing the testicular steroidogenesis disturbance. Recent findings indicate that inflammation, OS, and ER stress are localized to specific organelles, such as mitochondria, ER, and the nucleus. During the UPR production in the ER, enzymatic components like protein disulfide isomerase, ER oxidoreductin-1, and Nicotinamide Adenine Dinucleotide Phosphate Hydrogen oxidase complexes (e.g., Nox4) contribute to ROS formation. This leads to OS, underscoring their critical roles in ER stress (64). Additionally, PERK, a protein associated with GRP78, and GRP94 during ER stress, dissociates Nrf2 from Keap-1. Nrf2 is a key regulator involved in the synthesis of enzymatic and nonenzymatic antioxidants; thus, decreased Nrf2 levels can lead to diminished antioxidant defenses and increased OS. ER stress also triggers ROS production in mitochondria. ROS from the ER can affect calcium channels, causing the release of Ca²⁺ from the ER, which subsequently enhances mitochondrial ROS production. This initiates the Krebs cycle and oxidative phosphorylation in the electron transport chain. The release of cytochrome c can disrupt electron transport, alter mitochondrial membrane potential, and further promote ROS production, creating a cycle of OS and inflammation (65).

During inflammation, COX-2 is an inducible enzyme that converts arachidonic acid to PGE-2 leading to the generation of ROS as byproducts. ROS, such as hydrogen peroxide and superoxide anion, activate signaling pathways like nuclear factor-kappa B and activator protein-1. These pathways upregulate COX-2 expression, creating a feed-forward loop that amplifies OS and inflammation (7, 66).

Elevated malondialdehyde levels have been observed in testicular tissues exposed to BPs, correlating with reduced expression of steroidogenic enzymes such as StAR, CYP11A1, CYP17A1, 17β-HSD, CYP19, and 3β-HSD, as well as decreased testosterone levels (4, 42, 60, 67). These findings establish a strong relationship among inflammation, OS, and ER stress, suggesting that they are interconnected and may be, both causes and effects of cellular damage.

**Figure 2:**
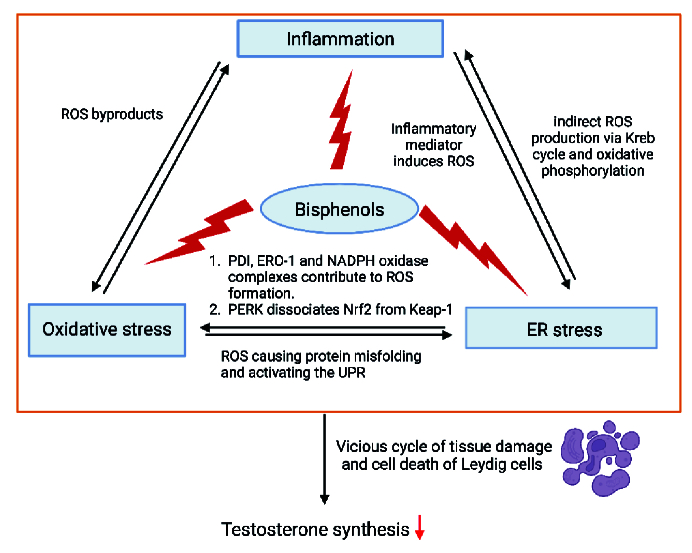
Proposed crosstalk between inflammation, oxidative stress, and ER stress. The interaction of inflammation, OS, and ER stress produces a feedback loop that intensifies cellular damage, including the disruption of testicular steroidogenesis. Cytokines and chemokines are examples of inflammatory mediators that can cause both OS and ER stress. ROS produced during OS might damage the ER, leading to improper protein folding and UPR activation. Consequently, this UPR activation triggers inflammatory pathways. It is hypothesized that if the inflammation process continued, it would eventually worsen OS and ER stress, resulting in a vicious cycle of tissue damage and cell death that lowers the ability of Leydig cells to synthesis testosterone. ER: Endoplasmic reticulum, ERO-1: ER oxidoreductin 1, Keap1: Kelch-like ECH-associated protein 1, NADPH: Nicotinamide adenine dinucleotide phosphate, Nrf2: Nuclear factor erythroid 2-related factor 2, OS: Oxidative stress, PERK: Protein kinase RNA-like ER kinase, PDI: Protein disulfide isomerase, ROS: Reactive oxygen species, UPR: Unfolded protein response.

Testicular steroidogenesis occurs in the mitochondria and smooth ER of Leydig cells, where energy production and mitochondrial membrane potential are vital for steroid biosynthesis. Mitochondria-associated membranes facilitate lipid translocation between the ER and mitochondria, which is essential for converting cholesterol into active steroid hormones (30, 68). During OS, ROS can damage Leydig cells mitochondria, reducing StAR expression and cholesterol transport, which in turn decreases testosterone synthesis (69). Additionally, inflammation, OS, and ER stress lead to lower levels of key steroidogenic enzymes like 3β-HSD, 17β-HSD, StAR, CYP11A1, and CYP17A1, resulting in decreased testosterone production and increased estradiol synthesis (70). The proposed interlinkage between inflammation, OS, and ER stress in BP-induced steroidogenesis disturbances are illustrated in figure 2.

**Table 4:** Bisphenol-induced ER stress evidence in testicular steroidogenesis from in vivo and in vitro models

**Author, year (Ref)**	**Type of bisphenol**	**Dosage**	**Duration of exposure**	**Animal model/cell line**	**Findings (ER stress)**	**Findings (steroidogenesis)**
**Qi ** ***et al.*** **, 2024 (42)**	BPA	10 μmol/L	24 hr	TM3 mouse Leydig cells	↑ GRP78, ↑ GRP94, ↑ p-PERK, ↑ p-IRE1, ↑ ATF6α, ↑ XBP1, ↑ GRP78	↓ StAR, ↓ CYP11A1, ↓ CYP17A1, ↓ 17β-HSD
**Yao ** ***et al.*** **, 2024 (41)**	TCBPA	25, 50, 75, and 100 μM	24 hr	TM3 mouse Leydig cells	↑ GRP78, ↑ eIF2α phosphorylation, ↑ ATF4	↓ Serum T ↓ Genes and protein expression of Scarb1, Cyp11a1, Cyp17a1, Hsd3b6, Hsd17b3
**Yin ** ***et al.*** **, 2017 (62)**	BPA	20, 40, or 80 μM 3, 30 mg/kg/d, and 300 mg/kg/d	24 and 48 hr 5 wk	Mouse spermatocyte-derived GC-2 cells Adult male Kunming mice	↑ GRP78, ↑ p-PERK, ↑ p-eIF2α, ↑ chop and ↑ ATF6 in a time- and dose-dependent manner; p-IRE-1, ↑ GRP78, p-PERK, p-eIF2α, chop, p-IRE1 and ATF6	NA
**Caglayan and Ozden 2023 (63)**	BPA, BPF, and BPS	0.1, 1, and 10 μM	48 hr	PNT1A and PC-3 human prostatic cell line	↑ Expression levels of ER stress-related genes, ↑ CHOP/DDIT3 in PNT1A	NA
↑ : Increased, ↓ : Decreased, μM: Micromolar, μmol/L: Micromoles per liter, mg/kg/d: Miligrams per kilogram per day, ATF4: Activating transcription factor 4, ATF6: Activating transcription factor 6, ATF6α: Activating transcription factor 6 alpha, BPA: Bisphenol A, BPF: Bisphenol F, BPS: Bisphenol S, CHOP: C/EBP homologous protein, chop: Gene encoding C/EBP homologous protein, CYP11A1: Cytochrome P450 11A1, Cyp11a1: Gene encoding cytochrome P450 11A1, CYP17A1: Cytochrome P450 17A1, DDIT3: DNA damage-inducible transcript 3, eIF2α: Eukaryotic initiation factor-2α, ER: Endoplasmic reticulum, GRP78: Glucose-regulated protein 78, GRP94: Glucose-regulated protein 94, h: Hour, Hsd3b6: Gene encoding hydroxy-delta-5-steroid dehydrogenase, Hsd17b3: Gene encoding hydroxysteroid 17-beta dehydrogenase 3, NA: Not applicable, p-eIF2α: Phosphorylated EIF2α, p-IRE-1: Phosphorylated inositol-requiring enzyme 1, p-PERK: Phosphorylated protein kinase RNA-like ER kinase, Scarb1: Gene encoding scavenger receptor class B type I, StAR: Steroidogenic acute regulatory protein, T: Testosterone, TCBPA: Tetrachlorobisphenol A, XBP1: X-box binding protein 1, 17β-HSD: 17-beta-hydroxysteroid dehydrogenase						

## 6. Conclusion

COX-2 and Nrf2 have been identified as key targets in BP-induced inflammation and OS, respectively, while ER stress also contributes to testicular steroidogenesis disturbances. Despite discussions on the connections between inflammation, OS, and ER stress in this context, recent insights into these associations are limited. It is noteworthy that most of these studies were carried out in animal models, even if the reported findings offer vital insights into the mechanisms and their therapeutic applications. Therefore, the translational value of these findings must be confirmed by additional validation through well-designed human studies. Therefore, future research should focus on the adverse effects of BPs on the interplay between inflammation, OS, and ER stress in testicular steroidogenesis to better understand the mechanisms behind BP-induced male reproductive toxicity. These findings highlight the complex interactions among these molecular pathways. Further studies are crucial to fully elucidate these mechanisms and develop effective interventions to safeguard reproductive health against environmental endocrine disruptors. These insights also emphasize the urgency of health policy measures to address BP exposure risks, including stricter regulations on BP usage and promoting safer alternatives. Additionally, the exploration of targeted therapies, such as COX-2 inhibitors, Nrf2 activators, and ER stress modulators, could pave the way for novel strategies to mitigate the reproductive toxicity caused by BPs.

##  Data Availability

Data supporting the findings of this study are available upon reasonable request from the corresponding author.

##  Author Contributions

Z. Abd Hamid, SB. Budin, and IS. Taib designed the study. IS. Taib, NES Jefferi, and AA. Shamhari conducted the research and drafted the manuscript. IS. Taib and NES. Jefferi monitored, evaluated, and analyzed the results of the study. Further, Z. Abd Hamid and SB. Budin reviewed the article. IS. Taib, NES. Jefferi, and AA. Shamhari reviewed the article and made the necessary corrections as requested by the referees. All authors approved the final manuscript and take responsibility for the integrity of the data.

##  Conflict of Interest

The authors declare that there is no conflict of interest.
